# Mathematical modelling and time series clustering of Mpox outbreak: A comparative study of the top 10 affected countries and implications for future outbreak management

**DOI:** 10.1016/j.gloepi.2025.100214

**Published:** 2025-07-27

**Authors:** Mark-Daniels Tamakloe, Ametus Kuuwill, Ibrahim Osumanu, Helina Siripi

**Affiliations:** a Division of Computational and Data Sciences, Mckelvey School of Engineering, Washington University in St. Louis, United States of America; b Forest Institutions and International Development (FIID) Research Group, Chair of Tropical and International Forestry, Faculty of Environmental Sciences, Technische Universität Dresden, Pienner Str. 7, 01737 Tharandt, Germany; c Department of Educational Psychology, University of Connecticut, United States of America; d Department of Economics Education, University of Education, Winneba, Ghana

**Keywords:** Mpox virus, Bootstrapping, Clustering, Dynamic Time Warping, Epidemics, Health shocks

## Abstract

The 2022 Mpox outbreak, characterized by its rapid cross-continental spread beyond traditionally endemic regions, presented a renewed threat to global health security. This study presents a comparative epidemiological analysis of the ten countries most affected by Mpox, integrating mathematical modelling with time series clustering, the first of its kind to analyze the 2022 WHO Mpox data. By applying an SIR-based model to estimate the effective transmission rate, basic reproduction number, time of first infection, and initial susceptible population, the study captures both the pace and persistence of Mpox spread, while critically assessing the effectiveness of national public health responses. Key findings reveal a paradox in North America: Canada exhibited a high transmission rate but a low reproduction number, indicating an elevated transmission potential per contact alongside limited secondary spread. This is likely due to concurrent containment measures or behavioral factors. In contrast, the United States, despite having a lower initial transmission rate, recorded a higher reproduction number. Similarly, Germany exhibited a similar risk trajectory, with elevated reproductive numbers despite robust infrastructure. The cases in the USA and Germany are likely due to systemic health and socio-political policy gaps and delayed behavior-targeted interventions, particularly in the population of men having sex with men (MSM). In Latin America, countries such as Peru and Mexico suffered disproportionately, likely due to limited access to healthcare, which compounded transmission dynamics and reproductive potential. Our study demonstrates that effective Mpox control is not solely dependent on health infrastructure, but also on behavioral targeting, equity, and adaptive health governance. This calls for cross-country and intercontinental collaborations towards combating current and future health shocks, including epidemics.

## Highlights

• Analyzed Mpox transmission dynamics in the top 10 most affected countries.
• Identified effective public health interventions, with Canada showing a lower reproduction rate.
• Highlighted the impact of healthcare disparities in Peru and Mexico on Mpox outcomes.
• Emphasized the need for global collaboration and targeted interventions for future outbreaks.

## Introduction

Infectious diseases, mostly of zoonotic origins remain a public health concern for both developing and so-called developed countries [1]. Some of these zoonotic infections include HIV/AIDS, Severe Acute Respiratory Syndrome (SARS) identified in China in 2002, and the Ebola virus outbreak [2]. Although not scientifically substantiated yet, commentators propagate the COVID-19 pandemic as a zoonotic infection [3,4]. COVID-19, as a global pandemic, has dominated the health literature since 2019, with keen focus on issues relating to the impact of vaccination and other quarantine measures [5,6]. This has however relegated attention to other life-threatening outbreaks, including the Monkeypox (Mpox) outbreak, described as a public health emergency by the World Health Organization [7]. The Mpox outbreak was first documented in 1958 in Central and West Africa and was found to belong to the Orthopoxvirus genus virology, which primarily targets animals like rats, squirrels, and Monkeys, among others [8,9,10,11]. As a result, Mpox transmission mediums has become a subject of scientific contention. Some studies holistically argue that Mpox is primarily transferred from animals to humans [12]. Yet, others argue that Mpox is not only transmitted from framed animals to humans but is also transmissible through human contact [13,1,14]. Investigations in this regard highlight that human-to-human Mpox transmission is possible through direct contact with an infected person’s body fluid, scabs, or infectious rash [15,16].

Yet, others argue that Mpox transmission is also possible through intimate physical contact, sexual activity^1^ and contact with respiratory secretions during extended direct communication with a human host [17,18,19]. While this debate is ongoing, recent studies turn to focus on the transmission and mortality dynamics of Mpox across different socioeconomic and sociocultural settings using different mathematical modelling approaches, yielding scientific parallels and convergencies. Using mathematical modelling, parameter estimation, and numerical simulations to analyze Mpox transmission dynamics, Olumuyiwa et al. [1] found that reducing transmission and progression rates significantly lowers Mpox cases and deaths. Hence, the study emphasized the need for policy effectiveness and targeted preventive measures against Mpox transmission in Nigeria and the Democratic Republic of Congo. Despite this rigorous investigation and plausible policy recommendation, the study could not highlight the reproduction and susceptibility rate of Mpox, limiting evidence-based policy interventions. Relying on classical and fractional-order differential equations, Peter et al. [11] established that adjusting control parameters, especially fractional orders, significantly influences the eradication of Mpox in Nigeria. Peter et al. [20] also used a deterministic mathematical model to investigate Mpox dynamics by considering two populations, humans and rodents. The study highlighted that it is economical and effective for Mpox prevention strategies to focus on preventing transmission from rodents to humans. Although this recommendation seems commendable, a fundamental question left unaddressed is the reproduction number and susceptibility rate between and among the two populations considered. This could have guided tailored policy recommendations given the recent threat of Mpox transmission from human-to-human, threatening life and global economic systems.

**Fig. 1:**
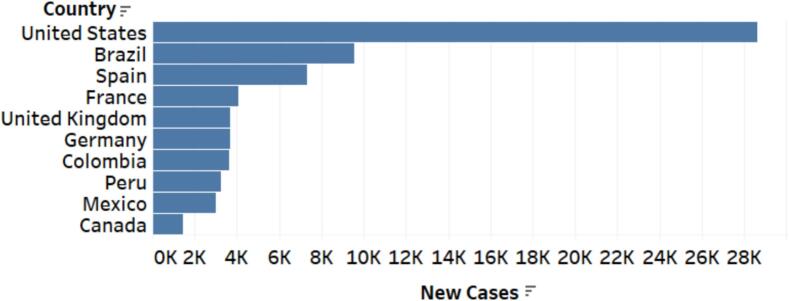
Total Cases of Mpox Outbreak Per Country ([7] data).

**Fig. 2:**
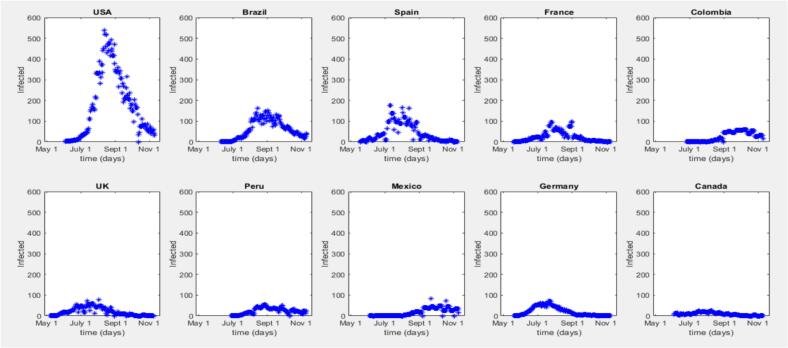
Time Series Progression for Mpox in the top 10 countries on the same scale. Note: Fig. 2 indicates an outbreak, but similarities are hard to discern on the same scale. Initial outbreak dates and peak levels vary by country. In Fig. 3, plotting each country's data on its own scale shows defined peaks, with the outbreak lasting longer at the peak in some countries. The U.S.A. and Germany have similar peak shapes, indicating a steady rise and fall, while Brazil and the U.K. have longer-lasting peaks.

**Fig. 3:**
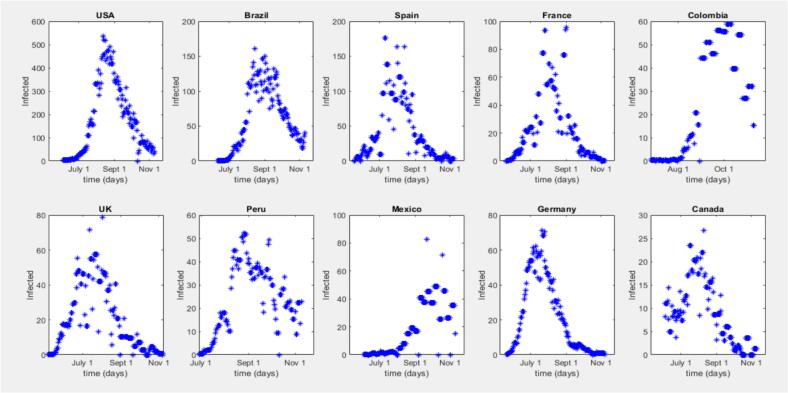
Time Series Progression for Mpox in the top 10 countries on the different scales.

The recent epidemiological shift wherein several non-African countries—historically peripheral to Mpox outbreaks—have become among the most severely affected countries has ignited concern for Mpox-related studies for public policy intervention. For instance, historically Mpox peripheral countries like the United States, Brazil, Spain, France, Colombia, the United Kingdom, Peru, Mexico, Germany, and Canada were the topmost hard-hit countries [7]. Available statistics show that the United States had the highest number of Mpox cases, totaling 28,379. Brazil followed with 9162 cases, while Spain had 7317 cases, and France, the UK, Germany, Colombia, Peru, and Mexico had comparable cases; however, Canada had the lowest total with 1437 cases (See Fig. 1, Fig. 2, Fig. 3). The World Health Organization (WHO) indicates no cure for Mpox hence, the need to pay critical attention to its spread and reproduction rate towards achieving good health and well-being (SGD3)([21].; [7]). In this light, Saldaña et al. [22] investigated instantaneous reproduction numbers and growth rates for the Mpox outbreak among European countries in 2022. According to the instantaneous reproduction numbers, the study found that Spain, France, Germany, the UK, Netherlands, Portugal and Italy need prompt policy responses.

Although this study established a scientific rigor in modelling recent Mpox transmission, solely relying on the instantaneous reproduction numbers or proxying the instantaneous reproduction numbers for the basic reproduction number represents a fundamental weakness. This is because the basic reproduction number represents the inherent transmissibility of a pathogen in a fully susceptible population, serving as a critical benchmark for assessing outbreak potential and intervention thresholds. Without this, it's hard to determine how much interventions have reduced transmission or what the disease's natural course would be without them. A global investigation by Bragazzi et al. [17] quantified the basic reproduction number and the underestimated fraction of mpox across 16 countries using Bayesian inference and a compartmentalized, risk-structured and two-route mathematical model. The study established that Mpox cases are significantly underreported, especially in countries like Colombia, with factors such as LGBTQ+ influencing the Mpox rate, calling for better surveillance and inclusive, community-based interventions targeting the subgroups of men having sex with one another (ibid). Despite the growing literature on Mpox, there is lack of comparative analyses among the topmost hit countries. This limits the global understanding of Mpox dynamics and constrains the development of context-specific interventions. Although Akhmetzhanov & Wu [23] estimated the basic reproduction number of Mpox in Mainland China, the studies failed to investigate the effective transmission rate and the Initial Susceptible Population, just like many other studies modelling Mpox dynamics [24,25,26,27]. Furthermore, the extensive literature have largely overlooked the effective transmission rate, time first infected and initial transmission rate.

The present study bridge these scientific gaps by investigating (1) the effective transmission rate, (2) the time first infected, (3) the initial susceptible population and (4) the basic reproduction number of Mpox, focusing on the top 10 affected countries as reported by the World Health Organization. The study employs a robust methodological framework, combining mathematical modelling with time series clustering, the first of its kind to combine these rigorous modelling approach to analyze cross-country variations in Mpox transmission dynamics. By focusing on susceptibility, effective transmission, and reproduction rates, the present study offers a scientifically rigorous and nuanced understanding of the Mpox spread, addressing a critical gap in comparative zoonotic disease research. Evidence in this context is crucial for implementing targeted surveillance, effective prevention strategies, and comprehensive control measures. The rest of the paper is presented as follows. The next section present the methodology section where the analytical rigor of the study is elaborated. The results and discussion follows this, leveraging on both deductive and inductive logic. This is followed by the study conclusion and policy recommendation.

## Methodology

### Mathematical modelling

Data for this work was sourced from the World Health Organization 2022 Mpox outbreak report, and mathematical modelling techniques were employed to estimate transmission dynamics across countries. Mathematical modelling helps simplify and analyze complex systems, predict future trends, and evaluate potential intervention strategies using mathematical equations and algorithms [28]. These models are extensively used in infectious disease epidemiology to assess interventions such as vaccination, isolation, and treatment [[29], [30]]. In this study, we focus on classical compartmental models, namely the Susceptible-Infected-Recovered (SIR) and the Susceptible-Exposed-Infected-Recovered (SEIR) models, to estimate the effective transmission rate, reproduction number, and initial conditions of Mpox outbreaks in the ten most affected countries.

The application of mathematical modelling to Mpox has grown substantially, with studies exploring both within-host dynamics and population-level transmission. For example, Deng et al. [31] modelled how different infection routes (e.g., intravenous, intradermal, and intrarectal) affect viral progression and immune responses, offering insight into biological mechanisms that inform control strategies. At the population level, models have been used to evaluate the impact of interventions during the 2022 outbreak in Canada, demonstrating how vaccination and isolation measures influenced the trajectory of infections across cities [32]. Other studies ([33]; Savinkina et al., [34] focus on optimizing vaccination strategies, particularly among high-risk populations such as men having sex with men (MHSM), incorporating behavioral and immunity dynamics into the modelling framework. More advanced modelling techniques have also emerged. These include fractional-order models to account for memory effects in disease transmission, and 7-dimensional compartmental models that capture multiple transmission routes and intervention effects. Optimal control frameworks have been employed to determine cost-effective intervention strategies under resource constraints. Predictive models combining mechanistic and time-series approaches have shown promise for early outbreak detection and planning. Our study contributes to this literature by using SIR/SEIR models calibrated to country-level data from WHO and applying bootstrapping and time series clustering to uncover cross-national outbreak dynamics—a relatively underexplored area in Mpox modelling.

The models used in this study, the SIR and SEIR frameworks are classical compartmental models widely adopted in infectious disease epidemiology [[29], [35]]. These are not new models but standard formulations applied to Mpox data from multiple countries to enable comparative estimation. The inclusion of an exposed compartment in the SEIR model is biologically justified by the virus's incubation period, typically ranging from 5 to 21 days, with most individuals developing symptoms within 21 days of exposure [10,11,7]. Accordingly, we assume a mean incubation duration of approximately 8 days, aligning with prior studies on Mpox latency [36]. For the recovery rate, we adopt a constant value γ=1/21, based on clinical data indicating that infected individuals generally recover within 2 to 4 weeks [37]. These parameter choices are consistent with prior modelling studies of Mpox ([10,11]; [38]) and are critical for ensuring biological plausibility in our simulations [39,40,41].

### The SIR model

**Fig. 4:**
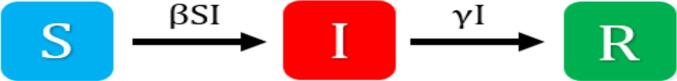
Compartmental diagram of the SIR model.

The SIR model is used to study and predict the spread of infectious diseases [42,43]. The model divides the population into three groups at any given time: susceptible (S), infected (I), and recovered (R). The model assumes the infection rate is proportional to the number of susceptible and infected individuals, while the recovery rate depends on the number of infected individuals [44]. It also follows the biological evidence substantiated rigorously by studies that once a person recovers from Mpox, such a person becomes immune and cannot be reinfected [45,46]. This is critical because understanding the effective transmission rate of Mpox helps identify how interventions like isolation or vaccination reduce spread. Knowing the time of first infection supports early detection systems to limit outbreaks. Estimating the initial susceptible population reveals immunity gaps that targeted vaccination can fill. Lastly, the basic reproduction number informs thresholds for herd immunity, guiding both current and future prevention strategies. The model's dynamics are illustrated in Fig. 4.

The set of differential equations that describe the SIR model is given by.

$$\frac{\mathrm{dS}}{\mathrm{dt}}=-\mathrm{\beta SI}$$

(1) $$\frac{\mathrm{dI}}{\mathrm{dt}}=\mathrm{\beta SI}-\mathrm{\gamma I}\;$$

$$\frac{\mathrm{dR}}{\mathrm{dt}}=\mathrm{\gamma I}.$$

Where βrepresents the effective transmission rate, and γ is the recovery rate. The total population denoted as N=S+I+Rremains constant over time because $\frac{\mathrm{dN}}{\mathrm{dt}}=0.$ The variables S, I,and Rindicate the number of people in each disease state and change over time. Typically, β is standardized by the population size N. However, for comparing the basic reproduction number $R_{0}$ between countries, we interpret it relative to each country's estimated initial population size to ensure context-specific comparability.

### The SEIR model

**Fig. 5:**
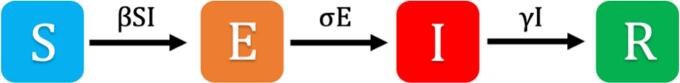
Compartmental diagram of the SEIR model.

The SEIR model is an expansion of the SIR model that incorporates an additional compartment $\left(E\right)$ to represent the time period during which individuals are exposed but not yet showing symptoms [47,48]. This model incorporates a time of latency in which an individual is infected but not yet symptomatic or capable of transmitting the virus, consistent with classical SEIR dynamics [36]. The SEIR model used in this study assumes the population is compartmentalized into four mutually exclusive groups: susceptible (S), exposed (E), infectious (I), and recovered (R) [49]. Individuals move from susceptible to exposed following contact with infectious individuals. The exposed compartment captures the biologically documented incubation period of Mpox, which ranges from 5 to 21 days, during which individuals are exposed to the virus, but not yet infectious [50,51]. This aligns with the natural history of the disease, where symptoms and transmission potential only emerge after this latent phase. It is further assumed that recovered individuals develop lasting immunity to Mpox, supported by the generation of virus-specific antibodies [45,46]. The model presumes homogeneous mixing in the population, which, while a simplification, enables analytical tractability in the absence of contact-network data. Fig. 5 illustrates the dynamics of the SEIR model.

The set of ordinary differential equations that describe the SEIR model is given by

$$\frac{\mathrm{dS}}{\mathrm{dt}}=-\mathrm{\beta SI}$$

(2) $$\frac{\mathrm{dE}}{\mathrm{dt}}=\mathrm{\beta SI}-\mathrm{\sigma E}\;$$

$$\frac{\mathrm{dI}}{\mathrm{dt}}=\mathrm{\sigma E}-\mathrm{\gamma I}$$

$$\frac{\mathrm{dR}}{\mathrm{dt}}=\mathrm{\gamma I}$$

Where β represents the effective transmission rate, σ is the incubation rate, γ is the recovery rate, and the total population N=S+E+I+R remains constant, with S,E,I,Rvarying over time.

### Inverse problems

Inverse problems aim to estimate the parameters of a mathematical or statistical model of a physical system based on observations of that system [52]. Instead of predicting the system's output given the parameters, inverse problems determine the parameters based on observed outputs [53,54]. In this paper, the Ordinary Least-Squares (OLS) method is used to estimate the parameters of the SIR model [55]. The statistical model assumes constant variance of the dependent (Y) variable, described by:

$$Y_{j}=f\left(t_{j};\theta_{0}\right)+\epsilon_{j},j=1,2,\ldots ,N$$

where $Y_{j}$ is a random variable for the observation at the time $t_{j},$ $f\left(t_{j};\theta_{0}\right)$ is the observed part of the solution of our statistical model with $\theta_{0}$ as the ‘true’ model parameters, and $\epsilon_{j}$  is the measurement error, assumed to be independent, identically distributed, with mean zero and constant variance. The OLS estimate for the parameter θ (is)

$$\theta_{O.L.S.}=\theta_{O.L.S.}^{N}\left(Y\right)=\arg\;\min_{\theta \in \Omega_{0}}\sum_{j=1}^{N}{\left|Y_{j}-f\left(t_{j};\theta \right)\right|}^{2}$$

In this paper, the aim is to estimate parameters β (the effective transmission rate), $t_{0}$(the initial time of the first infected case), and $S_{0}$  (the susceptible population). The recovery rate γ is assumed constant at $\frac{1}{21}$ per day, reflecting the mean infection period for Mpox. However, we assumed an initial infected individual at an unknown time $t_{0}$.The *fminsearch* algorithm in MATLAB is employed for parameter estimation using the Nelder-Mead simplex method. This method iteratively searches for the minimum of a function using a geometric approach with a simplex (a set of vertices). Table 1 provides the parameter estimates for the United States of America Mpox data, detailing the effective transmission rate (β) at $1.0740\times 10^{-4}\;$persons/day, the initial infection date ($t_{0}$) as May 31, 2022, the initial susceptible population ($S_{0}$ ) of 1389 people, and the basic reproduction number ($R_{0}$) of 3.28.

**Table 1:** Parameter Estimate for Mpox outbreak in the United States of America.

Parameter	Description	Estimate
β(1/person.day)	Effective Transmission Rate	$1.0740\times 10^{-4}$
$t_{0}$(days)	Time First Infected	May 31, 2022
$S_{0}$ (persons)	Initial Susceptible Population	1389
$R_{0}$(Reproduction Number)	Basic Reproduction Number	3.28

The assumption of constant variance is satisfied, indicated by a random scatter of points in the residual plot, suggesting normally distributed residuals with no systematic bias or trend. Following this, we estimated an initial model for all the topmost countries considered in the study. That is, we estimated the parameters β, $t_{0}$, $S_{0}$, and then calculate $R_{0}$ for the top ten Mpox countries using the formula $R_{0}=\frac{\beta S_{0}}{\gamma }\;$ , [56] where γ is fixed at $\frac{1}{21}$ . The β coefficient represents the effective transmission rate, with higher values indicating faster transmission. The $S_{0}$ value estimates the size of the susceptible population at the start of the epidemic and the $t_{0}$  value represents the estimated time of the first infection in each country. The basic reproduction number $R_{0}$ is a key threshold parameter in epidemiology, representing the average number of secondary cases generated by one infectious individual in a fully susceptible population [57]. Biologically, $R_{0}$ serves as a predictor of outbreak potential: if $R_{0}>1$, then the infection will likely spread through the population; if $R_{0}<1$, the outbreak is expected to die out over time. This leads to a fundamental result in infectious disease modelling:

Theorem 1 The disease-free equilibrium of the compartmental model is globally asymptotically stable if and only if $R_{0}<1$. Conversely, if $R_{0}>1$, the disease-free equilibrium becomes unstable, and the infection may persist for a while before eventually dying out.

This theorem is critical in public health because it provides a target for interventions: policies should aim to reduce the basic reproduction number below one. Measures such as vaccination, isolation, and behavior modification reduce transmission rates β, thereby lowering $R_{0}$ . In our context, countries with $R_{0}>1$, such as the United States and Germany, require sustained control efforts to prevent endemic spread, consistent with this theoretical framework.

**Fig. 6:**
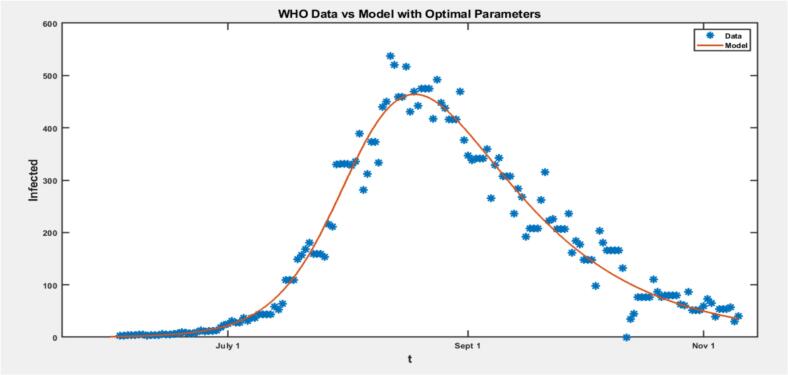
Graph of United States Mpox data with Optimal Parameters.

**Fig. 7:**
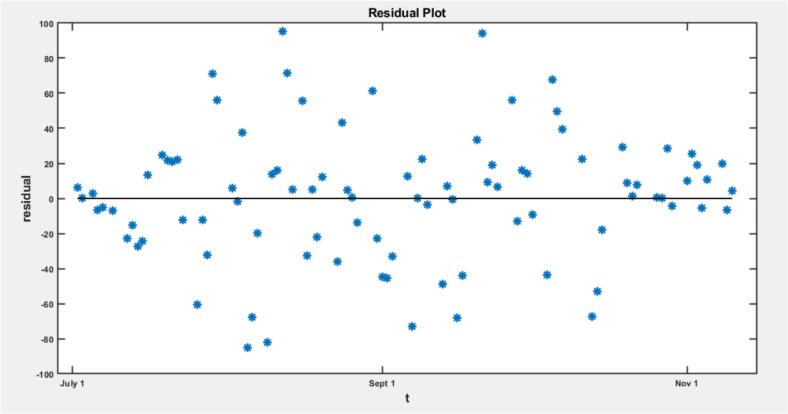
Residual Plot of United States Mpox data with Optimal Parameters.

The model fitting in Fig. 6 shows that the Mpox data averages the data effectively but fails to capture the infection peak, indicating that the peaks might be considered outliers by the model [42,43]. In Fig. 7, the plot of the residuals near the infection peak is higher, showing that the model fits well overall, excluding peak values, which the model considers as outliers [42,43]. The residual plot suggests that the assumption of constant variance is met, indicating normally distributed residuals around zero without systematic bias or trend. These observations highlight the model's limitations in accurately representing peak infection levels but confirm its general effectiveness for the overall data trend [42,43].

### Akaikie Information Criterion (AIC)

The Akaike Information Criterion (AIC) is a statistical tool that compares models based on their fit and complexity [58]. Introduced by Hirotugu Akaike in 1974, the AIC aims to balance the goodness of fit with the simplicity of the model [58,59,60]. It is calculated using the formula:

$$\mathrm{AIC}=-2\ln\left(L\left(\hat{\theta_{\mathrm{MLE}}}|y\right)\right)+2\kappa_{\theta }$$

where $\ln\left(L\left(\hat{\theta_{\mathrm{MLE}}}|y\right)\right)$ is the log-likelihood at its maximum value and $\kappa_{\theta }$ is the number of estimated parameters. For models estimated using the Ordinary Least Squares (OLS) method, the AIC under a constant variance model is:

$${\mathrm{AIC}}_{\mathrm{OLS}}=N\ln\left(\frac{\sum_{j=1}^{N}{\left[y_{j}-f\left(t_{j};\theta_{0}\right)\right]}^{2}}{N}\right)+2\left(\kappa_{\theta }+1\right)$$

AIC penalizes models with more parameters, preventing overfitting. The model with the lowest AIC is typically preferred because it strikes the best balance between fit and complexity. Akaike weights, calculated as:

$$w_{i}\left(\mathrm{AIC}\right)=\frac{e^{\left(-\frac{1}{2\;}\;\Delta_{i}\left(\mathrm{AIC}\right)\right)}}{\sum_{k=1}^{\kappa }e^{\left(-\frac{1}{2\;}\;\Delta_{k}\left(\mathrm{AIC}\right)\right)}}$$

Where κis the number of models, it helps to quantify the relative likelihood of each model being the best [59]. These weights are useful in multi-model comparison, allowing for a probabilistic assessment of model suitability. In comparing the SIR and SEIR models using AIC, the SIR model had an AIC of 1107.68 with a weight of 0.815, while the SEIR model had an AIC of 1110.65 with a weight of 0.185. Thus, the SIR model was chosen as the better model for this research due to its lower AIC and higher weight . Although the SEIR model accounts for latency, we adopted the simpler SIR model due to its parsimony and good empirical fit across countries. The SIR structure assumes individuals transition directly from susceptible to infectious, then to recover, which aligns with the observed dynamics where the latent period was short or data-limited. The model captures key public health levers: reducing transmission β or increasing recovery γ lowers the basic reproduction number $R_{0}$, thus shifting the system towards disease elimination. This formulation aligns with recent Mpox models that favor simplicity for real-time outbreak analysis (Malik & Althobaiti, 2025; [20]). The classical SIR model was selected for its simplicity, interpretability, and practical adequacy for modelling Mpox transmission dynamics using publicly available daily case counts. While more complex models (e.g., SEIR, fractional-order, or host-vector models) may offer additional granularity, they often require latent-period or reservoir-specific data, which were not uniformly available across countries. Mpox has a relatively short incubation period, and empirical studies show that the SIR model can sufficiently capture its transmission dynamics for comparative purposes [17,22]. Our focus on parsimony ensured consistent parameter estimation across heterogeneous settings while maintaining model tractability for cross-country analysis.

## Results and discussion

### Initial Model Results for All Countries

Table 2 highlights the dynamics of Mpox spread across different countries. Effective transmission rates and initial susceptible populations significantly influence the disease's propagation. The timing of initial infections and the basic reproduction number further elucidate the potential for spread and the effectiveness of early interventions. The results (Table 2) for the top 10 Mpox-affected countries provide a comprehensive insight into the dynamics of the disease spread and the effectiveness of the initial response to outbreaks. This is shown by the effective transmission rate (β), the initial susceptibility population (S_0_), the initial time of the first infection (t_0_), and the basic reproduction number ((R_0_). Although Canada is the lowest affected by Mpox In North America [61,62], our analysis, however, highlights that Canada exhibits the highest effective transmission rate of the Mpox disease at 13.3056 × 10^−4^ persons/day. This indicates a rapid spread within the initial stages. A possible explanation for this is that identifying and preventing the Mpox spread at an early stage presents significant challenges for public health authorities in general, corroborating the findings of [63]. This is probably because Mpox was first identified in Africa, making it an endemic disease to Africa [64]; hence, its extension to Canada presents new challenges to the Canadian health authority in terms of identification and preventive measures. Contrarily, the United States exhibits the lowest effective transmission rate at 1.0740 × 10^−4^ persons/day, although the country records the highest Mpox cases [65]. This may be attributed to a general preparedness with strategic preventive control mechanisms and taking information and valuable lessons from its peers like the United Kingdom and Germany, which were first hit by the outbreak before it was experienced in America. Additionally, the results show that Canada first experienced the Mpox disease on the North American continent on May 13, 2022, which may have taken the country by surprise and, hence, the possible high effective transmission rate. This may have propelled the United States health system to learn, adapt, and prepare for a possible extension into its borders, explaining the relatively lower transmission rate. The forthgoing indicates the need for cross-country and intercontinental collaboration, learning, and adaptive disease prevention and control mechanisms in the fight against public health outbreaks. While our findings seem to have contradicted Cahill [66], who holistically stated that the initial response of the USA public health system to the Mpox outbreak was inadequate in terms of deploying diagnostic tests, treatments, and vaccines, our studies converged in that significant federal policy changes implemented in August 2022, coupled with proactive sexual risk reduction behaviors among men having sex with men may have resulted in a gradual decline in new Mpox diagnoses by December 2022. In Europe, however, Germany and France recorded a high effective transmission rate of 8.0127 × 10^−4^ and 5.7474 × 10^–4,^ respectively. Contrary to America taking lessons from Canada, resulting in a lower comparative transmission rate, Germany experienced a higher transmission rate, although the country recorded the outbreak close to a month after the United Kingdom first experienced the outbreak on April 17, 2022. This indicates the need for stronger collaboration and learning between European health systems in fighting infectious disease transmissions at its early stages.

**Table 2:** Model Results for the Top 10 Mpox Countries.

Country	β($\times 10^{-4}$)1/person.day	$S_{0}$ persons	$t_{0}$ days (2022)	$R_{0}$
United States	1.0740	1389	May 31	3.28
Brazil	2.3356	508	May 27	2.61
Spain	3.6154	384	May 13	3.05
France	5.7474	219	May 20	2.77
United Kingdom	5.3102	208	April 17	2.43
Germany	8.0127	182	May 12	3.21
Colombia	6.9501	195	July 20	2.98
Peru	6.2642	181	June 12	2.49
Mexico	5.4011	211	July 10	2.51
Canada	13.3056	81	May 13	2.37

A similar pattern is observed for Peru and Colombia, the most affected countries in Latin America. This pattern substantiates our argument for intercontinental and intercountry collaboration and transfer of knowledge and experiences as a logical and plausible path towards preventing or controlling public health crises. The basic reproduction rate (R_0_), which is pivotal for understanding the potential for disease spread, suggests that the United States had the highest R_0_ at 3.28. This suggests a high spread rate as each infected individual would, on average, infect approximately 3.28 persons. This suggests that although the United States may have a lower effective transmission rate at the early stages due to its strategic control methods, the reproduction of the diseases is projected to increase without stringent control mechanisms. Conversely, the analysis projects Canada to have the lowest Mpox reproduction rate. A possible explanation is that the early outbreaks, leading to a high effective transmission rate, may have led to the adoption of a more robust and stringent Mpox control mechanism, which helped fight against the disease's spread later. In Europe, however, although Germany and France recorded high effective transmission rates, the reproductive rate estimate also suggests higher reproduction in these countries, unlike Canada, learning from its rapid initial stage outbreaks, leading to a low reproduction rate. This suggests the need for a more robust and focused intervention in the fight against Mpox in these countries. Despite the model's usefulness, we encountered several challenges during the analysis. Data inconsistency and limited reporting across countries restricted the granularity of our modelling. Moreover, parameter identifiability posed difficulties, as different parameter combinations often produced similar epidemic curves, complicating the estimation of true transmission dynamics.

**Fig. 8:**
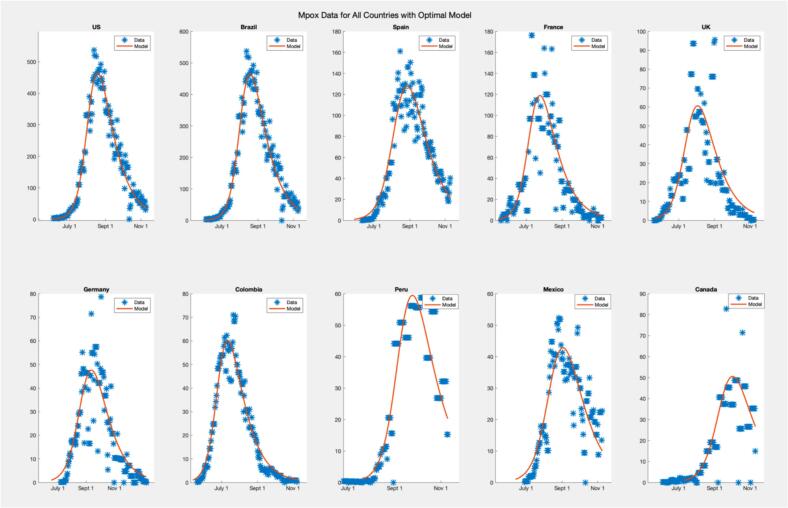
SIR Model Fits to 2022 Mpox Data for Top 10 Countries.

Fig. 8 compares models for different countries, including the United States, Brazil, Spain, France, the United Kingdom, Germany, Colombia, Peru, Mexico, and Canada. Each subplot displays the data and a model line plot for a specific country, facilitating a detailed comparison of model outputs for the top 10 countries. The models generally capture the outbreak trends well, but their peak values are often lower than the actual data peaks, highlighting the elimination of peak values from the modelling approach.

**Fig. 9:**
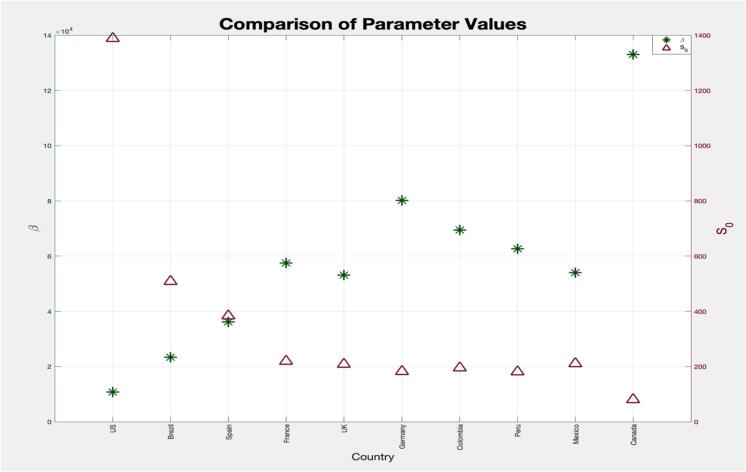
Comparison of Parameter Values for β and $S_{0}$ .

Fig. 9 compares parameter values for β and $S_{0}$  across the top 10 countries. Using dual y-axes, the left y-axis represents β, and the right y-axis represents $S_{0}$ , with countries arranged by prevalence. Green asterisks indicate β which are the values for the effective transmission rate, and red triangles indicate $S_{0}$ , representing values of the susceptibility rate. The graph highlights relationships between β and $S_{0}$  across countries, showing how population size might influence β. The USA shows a small β and a large $S_{0}$ , while Canada shows the opposite. This makes comparing outbreak similarities across countries difficult due to varying values. Hence, we included the $S_{0}$  in $R_{0}$  calculations, which helped to identify outbreak similarities across countries [36]. This method provides insights into transmission dynamics. The OLS method is sensitive to optimization algorithms like Nelder-Mead, which can fail without a unique minimum [67,68].

### Uncertainty Analysis and Bootstrapping

We performed nonparametric bootstrapping by repeatedly resampling the observed daily case data with replacement to generate 1000 synthetic datasets per country. For each resampled dataset, we re-estimated the key model parameters using the same optimization routine employed in the original fit. This generated a distribution of estimates for each parameter, from which we reported the 5th and 95th percentiles as the lower and upper bounds to reflect estimation uncertainty. This approach accounts for data variability and provides a robust basis for interpreting the stability and reliability of the inferred epidemiological parameters across countries.

Tables 3 show the median bootstrap parameter estimates for β, $S_{0}$ , $t_{0}$, and $R_{0}$ . These estimates include 90 % bootstrap intervals, calculated based on the 5 % and 95 % quantiles, providing plausible ranges for each parameter. This method accounts for uncertainties in our estimates. The results in Table 3 underline the variability and uncertainty inherent in our epidemiological modelling. The adjusted parameter estimates from the bootstrapping analysis provide a more comprehensive understanding of the Mpox transmission dynamics, highlighting the importance of considering uncertainty in model predictions. For example, the transmission rate in the United States was estimated at 1.029 × 10^−4^ persons/day, with a susceptible population of 1417. The reproduction rate was found to be 3.21. This suggests a slightly lower transmission rate compared with our initial estimate but still indicates a significant transmission potential. Brazil's transmission rate was 2.233 × 10^−4^ persons/day, with a susceptible population of 52, resulting in a reproduction rate of about  2.56. Upon further analysis, this lower Mpox reproduction rate compared to the initial estimate suggests a somewhat reduced transmission intensity. In Spain, the bootstrapping results indicated a transmission rate of 2.999 × 10^−4^ persons/day and a susceptible population of 422. Calculating the reproduction rate with uncertainty yielded 2.79, slightly lower than the initial estimate (See Table 2) but still reflecting a substantial spread. France showed a transmission rate of 4.155 × 10^−4^ persons/day, with a susceptible population of 262. The reproduction rate with uncertainty is estimated at 2.40, indicating a reduced transmission potential compared to the initial estimate. The United Kingdom's transmission rate was 4.424 × 10^−4^ persons/day, with an initial susceptible population of 232. The reproduction rate was recalculated to be 2.25, suggesting a lower transmission intensity than initially estimated. In Germany, the transmission rate was estimated at 7.966 × 10^−4^ persons/day, with an initial susceptible population of 181. The reproduction rate remained high at 3.18, consistent with the initial analysis. This suggests that Germany is likely to experience rapid Mpox transmission if sufficient public health policies are not adopted. Colombia's transmission rate was 6.662 × 10^−4^ persons/day, with an initial susceptible population of 198. The reproduction rate was 2.90, slightly lower than initially estimated but still indicating significant Mpox transmission. Peru's bootstrapping results showed a transmission rate of 5.744 × 10^−4^ persons/day, with an initial susceptible population of 190. The reproduction rate was recalculated to be 2.40, suggesting reduced transmission intensity. In Mexico, the transmission rate was estimated at 4.471 × 10^−4^ persons/day, with an initial susceptible population of 235 and a reproduction rate of 2.32, reflecting a lower transmission potential compared to the initial estimate. Canada's transmission rate was 11.865 × 10^−4^ persons/day, with an initial susceptible population of 87. The reproduction rate was 2.27, indicating a significant transmission potential but slightly lower than initially estimated.

**Table 3:** Median Parameter Estimates for the Top 10 Mpox Countries.

Country	β($\times 10^{-4}$)1/person.day	$S_{0}$ persons	$t_{0}$ days (2022)	R_0_
United States	1.029	1417	May 29	3.21
Brazil	2.233	521	May 25	2.56
Spain	2.999	422	May 3	2.79
France	4.155	262	May 2	2.40
United Kingdom	4.424	232	April 6	2.25
Germany	7.966	181	May 11	3.18
Colombia	6.662	198	July 17	2.90
Peru	5.744	190	June 8	2.40
Mexico	4.471	235	July 28	2.32
Canada	11.865	87	May 8	2.27

These results underline the variability and uncertainty inherent in epidemiological modelling. The adjusted parameter estimates from the bootstrapping analysis provide a more comprehensive understanding of the Mpox transmission dynamics, highlighting the importance of considering uncertainty in model predictions. The overall trends remain consistent with the initial analysis, affirming the significant transmission potential of Mpox in the top affected countries. In North America, Canada exhibits the highest transmission rate, indicating a potential for rapid spread in the initial stages. However, the lower reproduction rate suggests effective control measures are in place, reducing the long-term spread potential. The United States, with a lower transmission rate, shows a higher reproduction rate, indicating a significant spread potential if control measures are not maintained. The estimate of uncertainty streamlines Germany and the United States of America as the hotspot areas demanding cautious, strategic, and focused Mpox control interventions. Unsurprisingly, Germany is described as the economic hub of Europe, while the United States also remains the economic giant in North America with significant global influence [69,70,71]. These derive high populations in these countries, explaining why the Mpox reproduction rate is likely higher if not stringently controlled. Deductively, this suggests that Mpox spread in these countries significantly takes the form of human-to-human interaction, with one of the significant medium being men having sex with men (MSM) synonymously used as men having sex with one another. This suggest that liberalism, and the use of the term “freedom” to justify some socio-behavioral patterns have its own consequences, with its health implication being significant in this context.

In Latin America, Colombia has the highest transmission rate, with a reproduction rate of 2.90, indicating a substantial potential for rapid spread. Peru and Brazil also show notable transmission rates but with lower reproduction values, suggesting moderate control of the spread. These findings reinforce the need for tailored public health strategies based on specific country dynamics. Early detection, rapid response, and effective containment remain crucial. The variability in the transmission rates and reproduction numbers across countries highlights the importance of localized and adaptive strategies in managing infectious disease outbreaks. Collaborative efforts within and between countries are essential for effective disease control and prevention.

Further, the estimated bootstrap intervals for the top 10 Mpox-affected countries (Table 4) offer a nuanced understanding of the variability and uncertainty in the model parameters. These intervals provide insights into the possible ranges of the effective transmission rate, the initial susceptible population, the time of first infection, and the reproduction rate. The relatively narrow confidence intervals for the United States indicate high precision in the estimates, substantiating our early argument that Mpox is likely to spread with high intensity in the United States of America if not strategically tackled. Brazil shows moderate precision in its estimates. Compared to the United States, the broader range of effective transmission rates and the transmission number suggests more variability in the transmission dynamics, potentially due to regional differences in response and reporting. On the other hand, Spain has wider confidence intervals, especially for the effective transmission rate, indicating greater uncertainty in the transmission rate. The broad range for the infection time  suggests variability in detecting the initial cases, possibly due to differences in public health infrastructure or reporting delays. This calls for active health infrastructure development and resilient systems that could engage in rapid preventive and control actions against health crises.

**Table 4:** Estimated Bootstrap Intervals for the Top 10 Mpox Countries.

Country	90% CI for β($\times 10^{-4}$)	90% CI for $S_{0}$	90% CI for $t_{0}\;$(2022)	90% CI for $R_{0}$
United States	[0.973, 1.084]	[1390, 1450]	[May 25, June 1]	[3.10, 3.33]
Brazil	[2.074, 2.432]	[501, 539]	[May 19, May 31]	[2.46, 2.69]
Spain	[2.433, 3.833]	[383, 464]	[April 18, May 17]	[2.46, 3.23]
France	[2.790, 6.430]	[217, 327]	[March 30, May 26]	[2.00, 3.06]
United Kingdom	[3.673, 5.310]	[211, 257]	[March 23, April 18]	[2.07, 2.48]
Germany	[7.443, 8.618]	[176, 186]	[May 8, May 15]	[3.03, 3.35]
Colombia	[5.719, 7.780]	[187, 213]	[July 10, July 25]	[2.65, 3.25]
Peru	[4.970, 6.639]	[177, 205]	[May 30, June 16]	[2.24, 2.59]
Mexico	[1.789, 7.892]	[176, 429]	[May 1, July 28]	[1.71, 3.12]
Canada	[9.821, 14.805]	[78, 97]	[April 28, May 19]	[2.08, 2.55]

France exhibits significant variability in the effective transmission rate and susceptibility  population, reflecting uncertainty in the transmission rate and the initial susceptible population. The wide range for the infection time  indicates potential delays or inconsistencies in identifying the first infection, highlighting challenges in the initial response. The United Kingdom and Germany show moderate variability, with the transmission rate and initial susceptible population having relatively tight ranges. The early infection time  range suggests that the outbreak was identified quickly, possibly due to robust public health surveillance. Colombia's wider intervals suggest effective transmission rate variability in transmission dynamics. The late infection time range reflects a later onset of the outbreak compared to other countries, which might have influenced the public health response. Peru shows moderate variability, with slightly broader intervals for βand $S_{0}$. The variability in $t_{0}$  suggests some delay in identifying the initial cases, impacting the initial response strategy. Mexico's broad confidence intervals for all parameters indicate significant uncertainty in the estimates. The wide range for $t_{0}$  suggests considerable variability in the detection and reporting of initial cases, reflecting potential challenges in public health infrastructure. Canada shows high transmission rate variability β but a narrow range for $t_{0}$, indicating efficient early detection. The narrow range for $R_{0}$ suggests effective control measures once the outbreak is identified. The result suggests that Germany and Canada will likely experience higher and faster Mpox spread due to their high transmission rates, despite their effective initial detection and control measures. In contrast, Mexico shows significant uncertainty, reflecting potential challenges in managing the outbreak. The variability in β and $t_{0}$  across countries highlights the importance of robust public health infrastructure and rapid response strategies to control infectious diseases effectively.

### Time Series Clustering

**Fig. 10:**
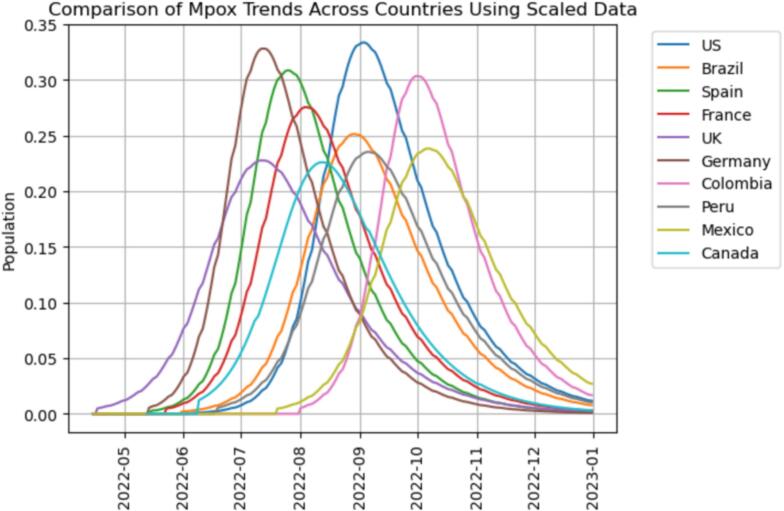
Comparison of Mpox Trends Across Countries.

**Fig. 11:**
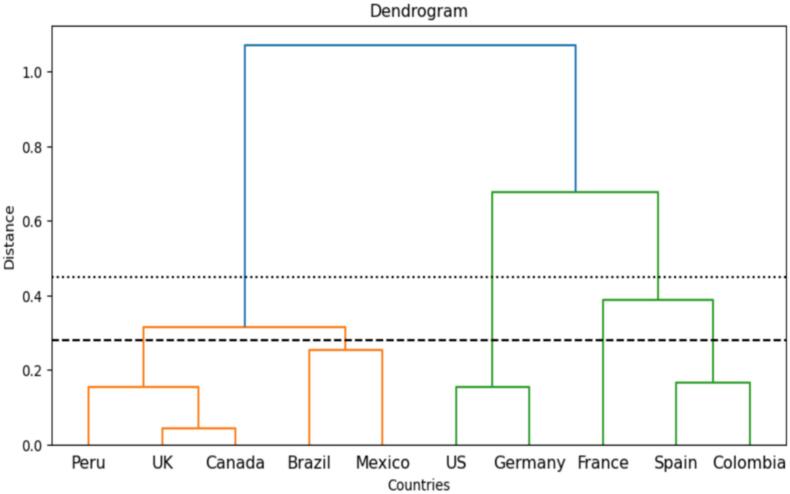
Dendrogram of Countries Based on Time Series Data.

Further, we used time series clustering to analyze the Mpox outbreak data to identify similarities and differences in the outbreak patterns among the top ten affected countries. This is based on the principle that the time series clustering technique groups similar time series data into clusters based on similarities and trends (Meesrikamolkul et al., 2012). In doing this, we adopted the Dynamic Time Warping (DTW) and hierarchical clustering approach as the distance metric on the top 10 countries affected by Mpox [72,73]. For our purposes, the mathematical model already gives an average trend for the outbreak (recall Fig. 8); therefore, we used the model to generate a smoothed estimate for the daily outbreak. Due to the inverse-like relationship between β and $S_{0}$ for some countries, we scaled the data by dividing each country's model-simulated data by $N=S_{0}+I_{0}+R_{0}=S_{0}+1$ where values of $S_{0}$ are given in Table 2. This N refers to the effective modeled population at the outbreak onset, not the entire national population. This helped us to get the overall dynamics of the Mpox virus [72]. We further assumed 0 infected population until the respective dates given by $t_{0}$ for the country. Fig. 10 shows the comparison of Mpox trends across countries using the scaled data. It is important to note that the population sizes are now expressed relative to each country's overall population N. Consequently, the y-scale in the graph represents a percentage or proportion of the total population, with infected individuals starting at $\frac{1}{N}$   for each country. This adjustment allows for consistent comparisons and interpretations across different populations, considering the varying population sizes (ibid). (See Fig. 11.)

The results indicates that Germany and the United States of America seems to have the same pattern. Lending weight to our earlier position, Initial cases in both countries were often linked to travelers from regions where the disease is endemic. Further, studies indicates that these countries have a higher prevalence among certain social networks, particularly within communities of men who have sex with men (MSM) [74,75]. While MSM in these two countries could possibly explain this [76,77], the two countries also have share historical, cultural, and linguistic ties, leading to strong social connections and frequent travel between the two countries [33]. Studies indicates similarities between the United Kingdom and Canada health reforms [78,79], explaining why they may have fallen in same cluster. The early stages of the spread may have taken these two countries by surprise; however, our results shows that they have resilience in strategic long term Mpox combating policies, reducing the reproduction rate of the virus. This reaffirms our early position of cross-country learning and strategic investment in health care system towards a more resilient disease outbreak prevention or control mechanisms. Peru and Mexico have large urban populations concentrated in major cities like Lima and Mexico City. Hence, high population density in these urban centers facilitates close human contact, which is a key factor in the transmission of Mpox, possibly explaining why these two countries fall into the same cluster [80]

### Dynamic Time Warping (DTW)

Also, we used the DTW algorithm to model-simulated data for the Mpox outbreak in the top ten countries to cross-validate our results. DTW was chosen because it effectively captures similarities between time series that may vary in timing but share underlying shape dynamics. This is especially relevant for epidemic curves that differ in peak periods and growth timing across countries. Unlike Euclidean distance, DTW aligns sequences non-linearly, making it robust to temporal shifts, and thus more appropriate for clustering heterogeneous outbreak trajectories. DTW is a technique used to compare the similarity of two sequences that may differ in length or pace [81]. A distance matrix was constructed to represent the pairwise DTW distances between each pair of countries. This matrix served as the basis for further hierarchical clustering analysis. Following the DTW algorithm, a 10 × 10 distance matrix was created for the top 10 mpox model-simulated data. This distance matrix is crucial for hierarchical clustering and represents pairwise distances between the dataset's series. The Python method calculates DTW distances between each series pair by iterating through the series indices and executing the DTW algorithm, excluding self-comparisons. The computed distances are recorded in the distance matrix, which is essential for clustering, allowing the identification of similarity patterns and hierarchical relationships. Table 5 displays the distance matrix output. Each cell indicates the degree of similarity or dissimilarity between the two countries. The DTW algorithm measures these distances by aligning the elements of the time series. Lower distance values signify higher similarity, while higher values denote greater dissimilarity. For example, the distance between the United States and Brazil is 0.34, showing moderate dissimilarity. In contrast, the distance between the U.K. and Canada is only 0.02, indicating high similarity

**Table 5:** DTW Distance Measure for the Top Ten Mpox Countries.

Country	U.S.	Brazil	Spain	France	UK	Germany	Colombia	Peru	Mexico	Canada
U.S.	0.00	0.34	0.09	0.22	0.47	0.07	0.10	0.42	0.41	0.47
Brazil	0.34	0.00	0.21	0.08	0.08	0.29	0.18	0.05	0.08	0.09
Spain	0.09	0.21	0.00	0.11	0.32	0.06	0.09	0.29	0.31	0.33
France	0.22	0.08	0.11	0.00	0.18	0.18	0.11	0.15	0.19	0.19
UK	0.47	0.08	0.32	0.18	0.00	0.41	0.30	0.05	0.16	0.02
Germany	0.07	0.29	0.06	0.18	0.41	0.00	0.12	0.37	0.40	0.42
Colombia	0.10	0.18	0.09	0.11	0.30	0.12	0.00	0.26	0.24	0.31
Peru	0.42	0.05	0.29	0.15	0.05	0.37	0.26	0.00	0.06	0.04
Mexico	0.41	0.08	0.31	0.19	0.16	0.40	0.24	0.06	0.00	0.14
Canada	0.47	0.09	0.33	0.19	0.02	0.42	0.31	0.04	0.14	0.00

### Hierarchical Clustering

**Fig. 12:**
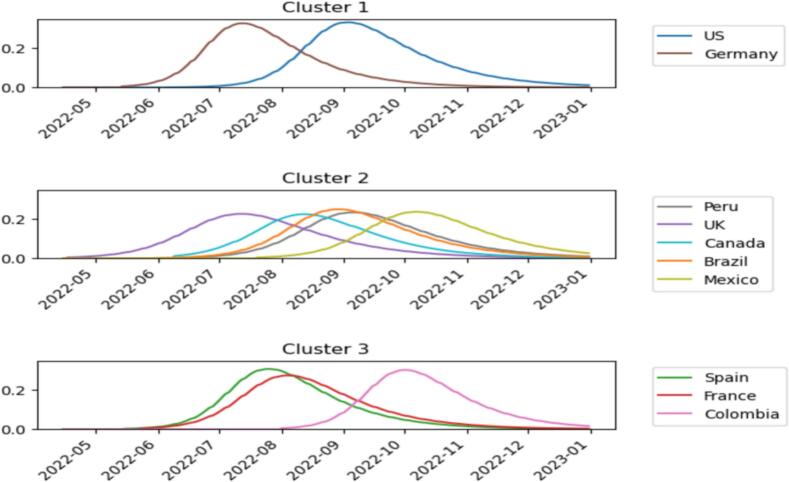
***Clustering Results on Scaled Mpox*** Data.

Following these insights, performed a hierarchical clustering using the DTW distance matrix. This method organizes the countries into clusters based on the similarity of their epidemic time series. The clustering revealed several insights. Countries with similar Mpox reproduction values $R_{0}$, such as the United States and Germany, were grouped together, indicating similar epidemic patterns, which reaffirmed our time series clustering results. Other countries with different reproduction values but similar time series patterns were also grouped together, highlighting nuances in the epidemic dynamics not captured by Mpox reproduction alone. The algorithm continues until all countries have been clustered. The dendrogram in Fig. 11 shows the linkages, confirming our results, with the United Kingdom and Canada in the same group. The dendrogram illustrates the hierarchical relationships among clusters, where each leaf node represents a country, and the branches show the merging process [82]. The branch heights indicate the distances between clusters. The graph examines our time series data's complete linkage clustering approach (ibid). The dendrogram reveals similarities and the hierarchical structure of the countries. By counting the vertical branches (cluster merges) intersecting the line at a height of 0.45, we identify the number of clusters formed at this height. We observe three clusters at this threshold, as shown in Fig. 12. The time series data analysis from the top 10 Mpox-affected countries reveals three distinct clusters. These clusters help understand the dynamics of the outbreak across different nations. The clustering analysis highlights similarities in outbreak patterns across different countries. For example, countries clustered together may share common public health measures, population behaviors, or socioeconomic factors.

The United States and Germany constituted the first cluster. These countries share several underlying characteristics influencing Mpox cases, as we have detailed above. This indicates that the United States and Germany stand a high risk of Mpox reproduction if strong policy interventions are not adopted. The second cluster from this analysis consists of Peru, the United Kingdom, Canada, Brazil, and Mexico. In addition to our assertion earlier, these countries may show similar Mpox patterns due to their centralized healthcare systems, high urbanization levels, similar public health responses, socioeconomic conditions, cultural attitudes towards health, and comparable climate factors. These shared characteristics may have contributed to comparable outbreak dynamics across these countries. Spain, France, and Colombia may exhibit similar Mpox patterns due to factors such as widespread international travel, similar social behaviors, public health infrastructure, and governmental policies on disease management. The frequent movement of people between these countries. This corroborates our earlier assertion using time series clustering. Additionally, at a lower threshold (0.28), five clusters were identified: Cluster 1 includes the United States and Germany; Cluster 2 consists of Brazil and Mexico; Cluster 3 comprises the United Kingdom, Peru, and Canada; Cluster 4 contains Spain and Colombia; and Cluster 5 is France, which exhibits a distinct pattern. Again, the United States and Germany emerged as the first cluster, substantiating our point that these countries are likely to be high-risk if strategic policies are not adopted. The clustering results primarily reflect differences in the relative peak magnitude and timing of outbreaks, with countries exhibiting high, medium, or low intensities tending to group together. The truncation levels (0.45 and 0.28) were selected by visually inspecting the dendrogram and observing stable groupings consistent with know epidemiological patterns.

## Conclusion, policy implication and future research path

Although the literature on Mpox transmission dynamics has grown over the past years, comprehensive study estimating the Mpox epidemiological parameters such as reproduction number, effective transmission rate, time first infected, and initial susceptible population (S_0_) is lacking. This study bridges this gap by focusing on ten of the most affected countries using 2022 World Health Organization data. We paired these estimates with hierarchical clustering, which enabled us to examine not only how Mpox spread over time but also how effectively public health systems responded across diverse epidemiological and socio-political contexts. Our key finding lies in the paradoxical dynamic within North America. The results revealed that Canada recorded a high effective transmission rate (β) and an early-time infection (t_0_). This indicates intense person-to-person contact at the onset, possibly due to low initial awareness and delayed containment. Despite this, Canada maintained a relatively low reproduction rate. This suggests that robust public health interventions, which likely included targeted messaging for high-risk populations like men having sex with one another, early isolation protocols, and rapid testing, may have quickly curtailed the Mpox outbreak's progression. Canada's case highlights how early setbacks can be reversed through adaptive, inclusive, and evidence-based health strategies. On the contrary, the United States exhibited a lower β and slightly later t_0_ than Canada, but this was accompanied by a higher reproduction rate, underscoring lingering community transmission. While initial containment may have benefited from proximity to Canada's experience, the inability to sustain effective transmission control suggests weaknesses in ongoing risk communication, particularly among the community of men having sex with one another, fragmented federal-state coordination, and possibly health service fatigue. The persistent high R_0_ in a well-resourced setting reveals, like the United States indicates that well-established health infrastructure must be combined with behavioral engagement and sustained surveillance towards health shocks resilient societies.

In Europe, Germany's trajectory mirrored that of the United States, with moderate β and delayed t_0_ but a concerningly high R_0_. The high initial susceptible population (S₀) in densely populated urban centers may have further amplified local outbreaks. Germany's clustering with the U.S. suggests similar epidemiological vulnerabilities shaped by socio-behavioral rather than economic constraints. Latin America presents another layer of complexity. Countries like Peru and Mexico, despite having lower β values than Canada, were also among the earliest infected (low t_0_), with high R_0_ values and sizable S_0_ populations. This reflects both early exposure and insufficient health infrastructure. Their clustering with wealthier nations like the UK and Brazil underscores the multidimensional nature of disease vulnerability. It reinforces the idea that Mpox preparedness must go beyond income level and integrate behavioral risk factors, system responsiveness, and timely policy interventions. Comparatively, the results indicate that Germany (in Europe) and the United States (in North America) stand at high risk, possibly due to low-regulated socio-behavioral factors linked with communities of men having sex with one another. However, the high risk of relatively small economies like Peru and Mexico can be attributed to poor health infrastructure, emphasizing the importance of equity in healthcare access and resources in these countries. These patterns demonstrate that Mpox transmission and control outcomes result from the interaction of biological timing (t_0_), contact dynamics (β), initial exposure scale (S_0_), and health system agility.

The results suggest that investments in local diagnostic labs, mobile surveillance teams, and frontline worker training can help mitigate current outbreaks while facilitating future outbreak preparedness in developing countries. However, socio-behavioral shifts through education, especially among men having sex with men communities, could help reduce linked epidemiologies like Mpox in the so-called developed economies like the United States and Germany. Also, the importance of timely, coordinated interventions, equitable healthcare access, and international collaboration cannot be overstated. Our findings align with known Mpox biological features. The estimated basic reproduction number and transmission rates are consistent with observed Mpox spread patterns, particularly among high-risk groups. The short infectious period assumed in the SIR model corresponds to clinical observations of rapid symptom onset and recovery. Furthermore, our use of publicly reported data ensures that parameter estimates such as recovery and infection rates reflect real-world epidemiological behavior. These biological consistencies substantiate the applicability of our simplified model to current Mpox outbreaks [45,46].As the world continues to grapple with emerging infectious diseases, robust strategies employed by countries that have effectively curtailed infections offer a valuable framework for future responses, emphasizing the need for a holistic approach that considers both public health and socioeconomic factors through cross-country and continental collaboration in buffering efforts to mitigate emerging infectious diseases. Future studies should examine how β and R_0_ vary between men having sex with men (MSM), bisexual and heterosexual populations. This would clarify the role of sexual networks in disease propagation and support more nuanced, targeted interventions. Additionally, incorporating individual-level data, spatial heterogeneity, and mobility patterns could enhance model precision. Expanding the analysis to include data beyond the 2022 WHO reports would improve generalizability. Future work may also consider integrating fractional-order dynamics, socio-behavioral factors, and real-time surveillance data to inform adaptive public health responses. This studies solely rely on the 2022 World Health Organization data of the 10 most affected countries. Hence, generalizing these results should be done with caution.

## CRediT authorship contribution statement

**Mark-Daniels Tamakloe:** Methodology, Formal analysis, Data curation, Conceptualization, Writing – original draft. **Ametus Kuuwill:** Formal analysis, Writing – review & editing, Writing – original draft. **Helina Siripi:** Writing – review & editing. **Ibrahim Osumanu:** Writing – review & editing.

## Ethical consideration

This research used secondary data from the World Health Organization; hence, no informed consent was needed and sorted accordingly.

## Funding

The research received no funding

## Declaration of competing interest

The authors declare that they have no known competing financial interests or personal relationships that could have appeared to influence the work reported in this paper.

Disclosure of interest

The authors report there are no competing interests to declare

## Data Availability

The data that support the findings of this study are available from the first author, MDT, upon reasonable request.

## Footnotes

1 Most especially, the community of men having sex with men

## References

[1] Olumuyiwa J.P., Oluwatosin B., Mayowa M.O., Omame A. (2024). Modelling the transmission of Mpox with case study in Nigeria and Democratic Republic of Congo (DRC). Computational Methods for Differential Equations.

[2] Barbiero V.K. (2020). Ebola: a hyperinflated emergency. Global Health: Science and Practice.

[3] Ghareeb O.A., Ramadhan S.A. (2021). COVID-19-a novel zoonotic disease: Origin, prevention and control. Pak J Med Health Sci.

[4] Kumar V., Pruthvishree B., Pande T., Sinha D., Singh B., Dhama K. (2020). SARS-CoV-2 (COVID-19): zoonotic origin and susceptibility of domestic and wild animals. J Pure Appl Microbiol.

[5] Teklu S.W. (2022). Mathematical analysis of the transmission dynamics of COVID-19 infection in the presence of intervention strategies. J Biol Dyn.

[6] Teklu S.W. (2024). Impacts of optimal control strategies on the HBV and COVID-19 co-epidemic spreading dynamics. Sci Rep.

[7] WHO (2022).

[8] Bunge E.M., Hoet B., Chen L., Lienert F., Weidenthaler H., Baer L.R. (2022). The changing epidemiology of human monkeypox—A potential threat? A systematic review. PLoS Negl Trop Dis.

[9] Kumar N., Acharya A., Gendelman H.E., Byrareddy S.N. (2022). The 2022 outbreak and the pathobiology of the monkeypox virus. J Autoimmun.

[10] Peter O.J., Kumar S., Kumari N., Oguntolu F.A., Oshinubi K., Musa R. (2022). Transmission dynamics of Monkeypox virus: a mathematical modelling approach. Modelling Earth Systems and Environment.

[11] Peter O.J., Oguntolu F.A., Ojo M.M., Olayinka Oyeniyi A., Jan R., Khan I. (2022). Fractional order mathematical model of monkeypox transmission dynamics. Phys Scr.

[12] Sharma E., Malhotra S., Kaul S., Jain N., Nagaich U. (2023). Unveiling the Mpox menace: Exploring the intricacies of a zoonotic virus and clinical implications. Diagn Microbiol Infect Dis.

[13] Mohapatra R.K., Singh P.K., Branda F., Mishra S., Kutikuppala L.V.S., Suvvari T.K. (2024). Transmission dynamics, complications and mitigation strategies of the current mpox outbreak: A comprehensive review with bibliometric study. Rev Med Virol.

[14] Pinto P., Costa M.A., Gonçalves M.F.M., Rodrigues A.G., Lisboa C. (2023). Mpox person-to-person transmission—where have we got so far? A systematic review. Viruses.

[15] Jezek Z., Szczeniowski M., Paluku K.M., Mutombo M., Grab B. (1988). Human monkeypox: confusion with chickenpox. Acta Trop.

[16] Usman S., Adamu I.I. (2017). Modelling the transmission dynamics of the monkeypox virus infection with treatment and vaccination interventions. Journal of Applied Mathematics and Physics.

[17] Bragazzi N.L., Iyaniwura S.A., Han Q., Woldegerima W.A., Kong J.D. (2024). Quantifying the basic reproduction number and underestimated fraction of Mpox cases worldwide at the onset of the outbreak. Journal of the Royal Society Interface.

[18] Durski K.N. (2018).

[19] Satapathy P., Shamim M.A., Padhi B.K., Gandhi A.P., Sandeep M., Suvvari T.K. (2024). Mpox virus infection in women and outbreak sex disparities: A Systematic Review and Meta-analysis. Commun Med.

[20] Peter O.J., Madubueze C.E., Ojo M.M., Oguntolu F.A., Ayoola T.A. (2023). Modelling and optimal control of monkeypox with cost-effective strategies. Modelling Earth Systems and Environment.

[21] Anuradha M., Rao K.R. (2023). Overview on Monkey Pox an Emerging Viral Infection. A Review of Literature. European Journal of Molecular & Clinical Medicine (EJMCM).

[22] Saldaña F., Daza-Torres M.L., Aguiar M. (2023). Data-driven estimation of the instantaneous reproduction number and growth rates for the 2022 monkeypox outbreak in Europe. PloS One.

[23] Akhmetzhanov A.R., Wu P.-H. (2024). Transmission potential of mpox in Mainland China, June-July 2023: estimating reproduction number during the initial phase of the epidemic. PeerJ.

[24] Díaz-Brochero C., Cucunubá Z.M. (2024). Epidemiological findings, estimates of the instantaneous reproduction number, and control strategies of the first Mpox outbreak in Latin America. Travel Med Infect Dis.

[25] Pekar J.E., Wang Y., Wang J.C., Shao Y., Taki F., Forgione L.A. (2025). Transmission dynamics of the 2022 mpox epidemic in New York City. Nat Med.

[26] Ward T., Overton C.E., Paton R.S., Christie R., Cumming F., Fyles M. (2024). Understanding the infection severity and epidemiological characteristics of mpox in the UK. Nat Commun.

[27] Zhang W., Zhang J., Liu Q.-H., Zhao S., Li W.-Q., Ma J.-J. (2025). Behavior changes influence mpox transmission in the United States, 2022–2023: Insights from homogeneous and heterogeneous models. PNAS Nexus.

[28] Bailey James E. (1998). Mathematical Modeling and Analysis in Biochemical Engineering: Past Accomplishments and Future Opportunities. Biotechnol. Progr..

[29] Siettos Constantinos I., Lucia Russo. (2013). Mathematical Modeling of Infectious Disease Dynamics. Virulence.

[30] White Peter J., Mark C. Enright (2010). Mathematical Models in Infectious Disease Epidemiology. Infect. Dis..

[31] Deng S.-Z., Liu X.-Y., Su J.-J., Xiang L., Chang L.-T., Zhu J.-J., Zhang H.-L. (2025). Epidemiological and cluster characteristics of dengue fever in Yunnan Province, Southwestern China, 2013–2023. BMC Infect. Dis..

[32] Xiu F. (2024). *Characteristics of the sexual networks of gay, bisexual, and other men who have sex with men and impact of past interventions on mpox transmission during the 2022 outbreak in Montréal, Toronto, and Vancouver (Canada)*.

[33] Cabanelas P., Manfredi L.C., González-Sánchez J.M., Lampón J.F. (2020). Multimarket competition and innovation in industrial markets: Spain and Colombia in comparative perspective. Journal of Business & Industrial Marketing.

[34] Savinkina A., Kindrachuk J., Bogoch I.I., Rimoin A.W., Hoff N.A., Shaw S.Y., Pitzer V.E., Mbala-Kingebeni P., Gonsalves G.S. (2024). Modelling vaccination approaches for mpox containment and mitigation in the Democratic Republic of the Congo. The Lancet Global Health.

[35] Okuh B., Edike C. (2024). Mathematical modelling of epidemiology dynamics. Innovative J. Sci..

[36] Kong L., Duan M., Shi J., Hong J., Chang Z., Zhang Z. (2022). Compartmental structures used in modelling COVID-19: a scoping review. Infect Dis Poverty.

[37] CDC (2023). The Center for Disease Control and Prevention(CDC) Domestic Mpox Response-United States, 2022-2023.

[38] Marques J.A.L., Gois F.N.B., Xavier-Neto J., Fong S.J., Marques J.A.L., Gois F.N.B., Xavier-Neto J., Fong S.J. (2021). Epidemiology compartmental models—SIR, SEIR, and SEIR with intervention. Predictive Models for Decision Support in the COVID-19 Crisis.

[39] Hajjo R., Abusara O.H., Sabbah D.A., Bardaweel S.K. (2025). Advancing the understanding and management of Mpox: insights into epidemiology, disease pathways, prevention, and therapeutic strategies. BMC Infect Dis.

[40] Sharif N., Sharif N., Alzahrani K.J., Halawani I.F., Alzahrani F.M., La Díez, I. D. T (2023). Molecular epidemiology, transmission and clinical features of 2022-mpox outbreak: A systematic review. Health Science Reports.

[41] Van Dijck C., Hoff N.A., Mbala-Kingebeni P., Low N., Cevik M., Rimoin A.W. (2023). Emergence of mpox in the post-smallpox era—a narrative review on mpox epidemiology. Clin Microbiol Infect.

[42] Cooper I., Mondal A., Antonopoulos C.G. (2020). A SIR model assumption for the spread of COVID-19 in different communities. Chaos, Solitons & Fractals.

[43] Nikitina A.V., Lyapunova I.A., Dudnikov E.A. (2020). Study of the spread of viral diseases based on modifications of the SIR model. Computational Mathematics and Information Technologies.

[44] Adamu H.A., Muhammad M., Jingi A., Usman M. (2019). Mathematical modelling using improved SIR model with more realistic assumptions. Int J Eng Appl Sci.

[45] Ganesan A., Arunagiri T., Mani S., Kumaran V.R., Kannaiah K.P., Chanduluru H.K. (2025). From pox to protection: understanding Monkeypox pathophysiology and immune resilience. Tropical Medicine and Health.

[46] Mazzotta V., Matusali G., Cimini E., Colavita F., Esvan R., Notari S. (2025). Kinetics of the humoral and cellular immune response up to one year from mpox virus infection. Clin Microbiol Infect.

[47] Poonia R.C., Saudagar A.K.J., Altameem A., Alkhathami M., Khan M.B., Hasanat M.H.A. (2022). An enhanced SEIR model for prediction of COVID-19 with vaccination effect. Life.

[48] Tolles J., Luong T. (2020). Modelling epidemics with compartmental models. Jama.

[49] Hunter E., Kelleher J.D. (2022). Understanding the assumptions of an SEIR compartmental model using agentization and a complexity hierarchy. Journal of Computational Mathematics and Data Science.

[50] Alvarez J.M.E., Acuña M.H., Arias H.F.G., Alvarado F.E.P., Ramírez J.J.O. (2024). Estimation of incubation period of mpox during 2022 outbreak in Pereira, Colombia. Emerg Infect Dis.

[51] Ponce L., Linton N.M., Toh W.H., Cheng H.-Y., Thompson R.N., Akhmetzhanov A.R. (2024). Incubation period and serial interval of mpox in 2022 global outbreak compared with historical estimates. Emerg Infect Dis.

[52] Morshed J., Kaluarachchi J.J. (1998). Parameter estimation using artificial neural network and genetic algorithm for free-product migration and recovery. Water Resour Res.

[53] Banks H.T., Hu S., Thompson W.C. (2014).

[54] Strang G. (1986).

[55] Cintrón-Arias A., Castillo-Chávez C., Betencourt L., Lloyd A.L., Banks H.T. (2008).

[56] Van den Driessche P. (2017). Reproduction numbers of infectious disease models. Infectious disease modelling.

[57] Muzembo B.A., Kitahara K., Mitra D., Ntontolo N.P., Ngatu N.R., Ohno A., Khatiwada J., Dutta S., Miyoshi S.-I. (2024). The basic reproduction number (R0) of ebola virus disease: A systematic review and meta-analysis. Travel Medicine and Infectious Disease.

[58] Akaike H. (2011). Akaike’s information criterion. International Encyclopedia of Statistical Science.

[59] Banks H.T., Joyner M.L. (2017). AIC under the framework of least squares estimation. Applied Mathematics Letters.

[60] Banks H.T., Joyner M.L. (2019).

[61] Navarro C., Lau C., Buchan S.A., Burchell A., Nasreen S., Friedman L. (2023). Effectiveness of one dose of MVA-BN vaccine against mpox infection in males in Ontario, Canada: A target trial emulation. MedRxiv.

[62] Ugwu C.L.J., Asgary A., Wu J., Kong J.D., Bragazzi N.L., Orbinski J. (2024). Geographical distribution and the impact of socio-environmental indicators on incidence of Mpox in Ontario. Canada MedRxiv.

[63] Amer F., Khalil H.E.S., Elahmady M., ElBadawy N.E., Zahran W.A., Abdelnasser M. (2023). Mpox: Risks and approaches to prevention. J Infect Public Health.

[64] Nachega J.B., Sam-Agudu N.A., Ogoina D., Mbala-Kingebeni P., Ntoumi F., Nakouné E. (2024).

[65] Walsh-Buhi E.R., Walsh-Buhi M.L., Houghton R.F. (2024). Mpox knowledge in the US: Results from a nationally representative survey. J Infect Public Health.

[66] Cahill S. (2023). Lessons Learned from the US Public health response to the 2022 mpox Outbreak. LGBT Health.

[67] Beliakov G., Kelarev A., Yearwood J. (2012). Derivative-free optimization and neural networks for robust regression. Optimization.

[68] Omay T., Corakci A. (2023). A Unit Root Test with Markov Switching Deterministic Components: A Special Emphasis on Nonlinear Optimization Algorithms. Computational Economics.

[69] Becker P. (2023). Germany as the European Union’s status quo power? Continuity and change in the shadow of the Covid-19 pandemic. J Eur Publ Policy.

[70] Lauk K.J. (2018). In Search of Germany.

[71] Vögele S., Ball C. (2019).

[72] Cai X., Xu T., Yi J., Huang J., Rajasekaran S. (2019). Dtwnet: a dynamic time warping network. Advances in Neural Information Processing Systems.

[73] Senin P. (2008). Dynamic time warping algorithm review. Information and Computer Science Department University of Hawaii at Manoa Honolulu, USA.

[74] Chow E.P.F., Samra R.S., Bradshaw C.S., Chen M.Y., Williamson D.A., Towns J.M. (2023). Mpox knowledge, vaccination and intention to reduce sexual risk practices among men who have sex with men and transgender people in response to the 2022 mpox outbreak: a cross-sectional study in Victoria. Australia Sexual Health.

[75] Pinnetti C., Mondi A., Mazzotta V., Vita S., Carletti F., Aguglia C. (2024). Pharyngo-tonsillar involvement of Mpox in a cohort of men who have sex with men (MSM): A serious risk of missing diagnosis. J Infect Public Health.

[76] Álvarez-Moreno C.A., Alzate-Ángel J.C., Ilich H., Bareño A., Mantilla M., Sussman O. (2023). Clinical and epidemiological characteristics of mpox: a descriptive cases series in Colombia. Travel Med Infect Dis.

[77] Sönmez İ., Riveros H.M., Folch C., Suñer C., Díaz Y., Alonso L. (2023). Egocentric sexual network analysis among gay and bisexual men who have sex with men with and without mpox infection. Sex Transm Infect.

[78] Adams T.L. (2020). Health professional regulation in historical context: Canada, the USA and the UK (19th century to present). Hum Resour Health.

[79] Wang T., McAuslane N., Liberti L., Gardarsdottir H., Goettsch W., Leufkens H. (2020). Companies’ health technology assessment strategies and practices in Australia, Canada, England, France, Germany, Italy and Spain: an industry metrics study. Front Pharmacol.

[80] Coq-Huelva D., Asián-Chaves R. (2019). Urban sprawl and sustainable urban policies. A review of the cases of Lima, Mexico City and Santiago de Chile. Sustainability.

[81] Tormene P., Giorgino T., Quaglini S., Stefanelli M. (2009). Matching incomplete time series with dynamic time warping: an algorithm and an application to post-stroke rehabilitation. Artif Intell Med.

[82] Farrelly C.M., Schwartz S.J., Amodeo A.L., Feaster D.J., Steinley D.L., Meca A. (2017). The analysis of bridging constructs with hierarchical clustering methods: An application to identity. J Res Pers.

