# Corrigendum to ‘Mathematical modelling and time series clustering of Mpox outbreak: A comparative study of the top 10 affected countries and implications for future outbreak management’ [Global Epidemiology 10 (2025) 100214]

**DOI:** 10.1016/j.gloepi.2025.100216

**Published:** 2025-09-22

**Authors:** Mark-Daniels Tamakloe, Ametus Kuuwill, Ibrahim Osumanu, Helina Siripi

**Affiliations:** a Division of Computational and Data Sciences, Mckelvey School of Engineering, Washington University in St. Louis, United States of America; b Forest Institutions and International Development (FIID) Research Group, Chair of Tropical and International Forestry, Faculty of Environmental Sciences, Technische Universität Dresden, Pienner Str. 7, 01737 Tharandt, Germany; c Department of Educational Psychology, University of Connecticut, United States of America; d Department of Economics Education, University of Education, Winneba, Ghana

Joyner Michele Lynn was contacted on July 24, 2024, to help draft the original article and accept co-authorship, as she had supervised the master's thesis that contributed to this article. She did not respond to the email, possibly an oversight. To avoid ethical issues of adding an author without their explicit consent, the authors proceeded without her. After the article was published online on July 27, 2025, Joyner Michele Lynn expressed that she would have loved to co-author the article. In the spirit of collegiality, maintaining professional relationships and fairness, all authors have since agreed to include Joyner Michele Lynn as a co-author, acknowledging her contribution as supervision. Below are the details along with the revised author order and affiliations:

Name - **Joyner Michele Lynn**

Affiliation - Mathematics and Statistics, East Tennessee State University, Johnson City 37,614, USA

Mark-Daniels Tamakloe^a^, Ametus Kuuwill^b^, Ibrahim Osumanu^c^, Helina Siripi^d^, **Joyner Michele Lynn**^e^

^a^Division of Computational and Data Sciences, Mckelvey School of Engineering, Washington University in St. Louis, United States of America

^b^Forest Institutions and International Development (FIID) Research Group, Chair of Tropical and International Forestry, Faculty of Environmental Sciences, Technische Universität Dresden, Pienner Str. 7, 01737 Tharandt, Germany

^c^Department of Educational Psychology, University of Connecticut, United States of America

^d^Department of Economics Education, University of Education, Winneba, Ghana

^e^Mathematics and Statistics, East Tennessee State University, Johnson City 37614, USA

The authors would like to apologise for any inconvenience caused.

DOI of original article: https://doi.org/10.1016/j.gloepi.2025.100214

